# Unveiling the potential of spirulina algal extract as promising antibacterial and antibiofilm agent against carbapenem-resistant *Klebsiella pneumoniae*: in vitro and in vivo study

**DOI:** 10.1186/s12934-024-02619-3

**Published:** 2025-01-05

**Authors:** Mohamed I. Selim, Tarek El-banna, Fatma Sonbol, Walaa A. Negm, Engy Elekhnawy

**Affiliations:** 1 Pharmaceutical Microbiology Department, Faculty of Pharmacy, Tanta University, Tanta, 31527 Egypt; 2 Department of Pharmacognosy, Faculty of Pharmacy, Tanta University, Tanta, 31527 Egypt

**Keywords:** Carbapenem-resistance genes, Biofilm, Multidrug resistance, Pneumonia, LC/MS, qRT-PCR

## Abstract

Carbapenem-resistant *Klebsiella pneumoniae* poses a severe risk to global public health, necessitating the immediate development of novel therapeutic strategies. The current study aimed to investigate the effectiveness of the green algae *Arthrospira maxima* (commercially known as *Spirulina*) both in vitro and in vivo against carbapenem-resistant *K. pneumoniae*. In this study, thirty carbapenem-resistant *K. pneumoniae* isolates were collected, identified, and then screened for their susceptibility to several antibiotics and carbapenemase production genes using PCR. Both *bla*_*KPC*_ and *bla*_*OXA-48*_ genes were the most predominant detected carbapenemase genes in the tested isolates. The phytochemical profiling of *A. maxima* algal extract was conducted using LC–MS/MS in a positive mode technique. The minimum inhibitory concentrations (MIC) of the algal extract ranged from 500 to 1000 µg/mL. The algal extract also resulted in decreasing the membrane integrity and distortion in the bacterial cells as revealed by scanning electron microscope. The bioactive compounds that were responsible for the antibacterial action were fatty acids, including PUFAs, polysaccharides, glycosides, peptides, flavonoids, phycocyanin, minerals, essential amino acids, and vitamins. Moreover, *A. maxima* algal extract revealed an antibiofilm activity by crystal violet assay and qRT-PCR. A murine pneumonia model was employed for the in vivo assessment of the antibacterial action of the algal extract. *A. maxima* showed a promising antibacterial action which was comparable to the action of colistin (standard drug). This was manifested by improving the pulmonary architecture, decreasing the inflammatory cell infiltration, and fibrosis after staining with hematoxylin and eosin and Masson’s trichrome stain. Using immunohistochemical investigations, the percentage of the immunoreactive cells significantly decreased after using monoclonal antibodies of the tumor necrosis factor-alpha and interleukin six. So, *A. maxima* may be considered a new candidate for the development of new antibacterial medications.

**Graphical Abstract:**

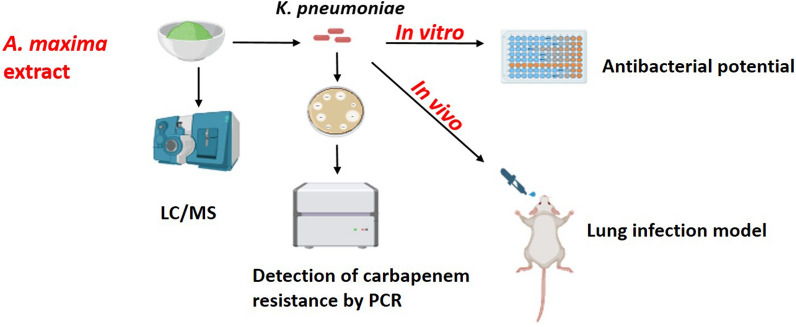

**Supplementary Information:**

The online version contains supplementary material available at 10.1186/s12934-024-02619-3.

## Introduction

In recent years, antibiotic resistance has become a significant issue that poses a challenge to medical personnel [1]. The multidrug-resistant microorganisms (superbugs) are almost resistant to most antibiotics available in the pharmaceutical market. Unfortunately, pharmaceutical companies cannot cope with the speedy spreading of multidrug-resistant bacteria by providing new drugs [2]. *K. pneumoniae* is an opportunistic, Gram-negative, non-motile, and enteric bacteria [3]. It is a part of the normal microbiome in healthy people residing in mucosal surfaces like the gastrointestinal tract and oropharynx but can spread to other tissues. It can trigger severe infections like pneumonia and other miscellaneous infections such as meningitis, septicemia, and purulent abscesses, especially in immunocompromised patients, newborns, or those at high risk in hospitals and clinics [4]. It is responsible for hospital-acquired infections, including pneumonia, urinary tract infections (UTIs), and bloodstream infections [5]. The extensive use of antibiotics contributed to the repeated outbreaks of multidrug-resistant *K. pneumoniae* [6].

Carbapenems are among the widely used antibiotics to treat *K. pneumoniae* infections. However, when they are overused or misused, they often contribute to the spreading of carbapenem-resistant *K. pneumoniae*. Three primary mechanisms are responsible for carbapenem resistance, and they are carbapenemase production, efflux pump, and porin alterations. However, the production of plasmid-mediated carbapenemase enzyme remains the essential mechanism of the pathogen’s resistance towards carbapenems [7]. The main genes responsible for carbapenemase production are bla-*K. pneumonia* carbapenemase (*bla*_KPC_), bla-oxacillin hydrolyzing enzymes-48 (*bla*_OXA-48_), bla-New Delhi metallo-β-lactamase (*bla*_NDM_), bla-Verona integron-mediated metallo-β-lactamase (*bla*_VIM_), and bla-active on imipenem (*bla*_IMP_) [8].

Among the new approaches to fight against antibiotic resistance using natural sources like green algae as antibacterials [9]. *Arthrospira maxima* (*spirulina*) is a blue-green algae (cyanobacterium) that has a commercial interest as a food supplement and it gained growing attention in recent years. It has several bioactive compounds which are suggested to be responsible for its antioxidant and immune-enhancing activities. These compounds include c-phycocyanin, a high protein content with all essential amino acids, essential fatty acids, minerals, pigments, carotenoids, flavonoids, and vitamins [10]. *A. maxima* has also shown various other pharmacologic effects like antibacterial [11], antifungal potential [12], and antiviral [13] actions. Its antibacterial action could be attributed to its bioactive substances like polysaccharides and phycocyanin, which break down bacterial cell walls, cause oxidative stress, and deprive cells of vital metals [14].

This study aimed to assess the antibacterial and antibiofilm activities of *A. maxima* extract against carbapenem-resistant *K. pneumoniae* isolates in vitro and in vivo using a pneumonia model in mice.

## Material and methods

### Identification of carbapenem-resistant *K. pneumoniae* and antibiotic susceptibility testing

#### Collection of carbapenem-resistant *K. pneumoniae* isolates

A total number of 30 clinical carbapenem-resistant *K. pneumoniae* isolates were recovered from different clinical samples collected from Mansoura University Hospitals, Mansoura International Hospital, and Cairo University Hospitals. Samples were drawn from blood, wound, urine, sputum, and pus as shown in Table S1. Recovered *K. pneumoniae* isolates were identified and tested for carbapenem resistance by antibiotic breakpoint detection. According to the Clinical and Laboratory Standards Institute (CLSI) 2020, the bacterial isolate is considered to be resistant if it had a minimum inhibitory concentration ≥ 4 μg/mL to meropenem. Isolates were then cultured in nutrient broth (Himedia, India) and stored in glycerol stock (50% glycerol/nutrient broth) at −80 °C until used for further studies.

#### Antimicrobial susceptibility testing

Kirby-Bauer disk diffusion technique was performed on Muller-Hinton agar (Himedia, India) for screening of the antimicrobial susceptibility [15, 16]. Meropenem (MEM, 10 µg) and imipenem (IPM, 10 µg) discs were used for confirmation of the presence of carbapenem resistance. Fifteen antibiotics representing different antibiotic classes were tested and they were colistin (CL, 10 µg), gentamicin (CN, 120 µg), amikacin (AK, 30 µg), cefazolin (CZ, 30 µg), cefuroxime (CXM, 30 µg), cefotaxime (CTX, 30 µg), ceftriaxone (CRO, 30 µg), ceftazidime (CAZ, 30 µg), cefepime (FEP, 30 µg), ciprofloxacin (CIP, 5 µg), levofloxacin (LEV, 5 µg), doxycycline (DO, 30 µg), piperacillin/tazobactam (TPZ, 100/10 µg), ampicillin/sulbactam (SAM, 10/10 µg), and cotrimoxazole (SXT, 23.75/1.25 µg).

#### Polymerase chain reaction (PCR)

*K. pneumoniae* isolates were subjected to PCR assay to detect the carbapenem resistance genes: *bla*_VIM_, *bla*_IMP_, *bla*_NDM-1_, *bla*_KPC,_ and *bla*_OXA-48_ [17] as previously reported (supplementary file). The primer sequence for the tested genes is shown in Table S2.

After completion of the process, the PCR product was allowed to run on agarose gel electrophoresis. The PCR products, the DNA ladder, and the negative control were loaded to 1.2% agarose gel and the power supply was set to 80 V for one hour. After completion, the gel was inspected for results on a UV transilluminator. Positive results were indicated by the detection of single sharp bands with a definite amplicon size for each gene.

### Preparation and characterization of *A. maxima*

#### Preparation of the extract

The blue-green algae *A. maxima* were obtained from Swanf Commercial and Trade Co., Ltd., China. *A. maxima* classification was carried out by Dr. Esraa Ammar at the Faculty of Science and it was assigned an ascension number 2022–01-PG-W-67. Classic cold maceration extraction was done using 100 g of dry *A. maxima* algal powder inside a closed container along with ethanol as a solvent for 72 h (three times, each one liter of ethanol) with stirring at regular intervals at room temperature. The mixture was then filtered, and the solvent was evaporated under vacuum at 40 °C. A rationale for ethanol solvent selection is this a polar solvent can enhance the yield of polyphenolic and flavonoid-based antibacterial agents.

For sample preparation for the phytochemical study, a one-milliliter solution of deionized water, methanol, and acetonitrile (50: 25: 25) was used to reconstitute a weighed part of the residue (50 mg). The dissolved sample was vortexed for two minutes, ultra-sonicated for 10 min, and centrifuged for another 10 min at 1000 rpm. To inject 10 µL of the sample solution at a concentration of 1 µg/µL, dilution was done using the reconstitution solvent. The remaining extract was kept in a refrigerator for further biological investigations.

#### Liquid chromatography-mass spectrometry (LC–MS/MS)

LC–MS/MS analysis of *A. maxima* extract was performed as reported before [18, 19]. The positive electrospray ionization approach was applied for the detection of the various phytoconstituents of the *A. maxima* extract and the targeted constituents were identified by comparing LC/MS data with previously published substances and reference databases [20–22].

### In vitro screening for the antimicrobial activity and antibiofilm action of *A. maxima*

#### Antibacterial potential of *A. maxima*

The broth microdilution method [23–25] was carried out to determine the minimum inhibitory concentration (MIC) of *A maxima*’s extract dissolved in 5% dimethyl sulfoxide (DMSO) against carbapenem-resistant *K. Pneumoniae* beginning with a concentration of 4000 μg/mL in the first well (highest concentration) of each row in a 96-well-microtiter plate. Serial two-fold dilution was performed till the tenth well (lowest concentration = 7.81 µg/mL) for the algal extract using nutrient broth as a positive control in the 11th well (the bacterial culture and nutrient broth) and negative control in the 12th well (nutrient broth only). A standard control row was used in which only a solution of 5% DMSO, which was used as a solvent for *A. maxima*, was added to the first well and two-fold serially diluted. After incubating of plates at 37 °C for 24 h, plates were inspected by ELISA reader (Robonik, India) at wavelength 630 nm. The MIC values were identified as the lowest concentration of algae at which no visible growth of bacteria after incubation of plates at 37 °C for 24 h. The test was repeated three times.

#### Impact of *A. maxima *on membrane properties of *K. pneumoniae*

Cell membrane integrity of* K. pneumoniae* was elucidated before and subsequent treatment with the algal extract (at 0.5 MIC) by observing the discharge of both DNA and RNA from the cells to the external medium [18, 26, 27]. After centrifugating overnight bacterial suspensions, the formed pellets were resuspended in sodium chloride solution (0.5%) followed by measuring absorbance at 260 nm using a UV/Vis spectrophotometer (SHIMADZU, Japan).

#### Scanning electron microscope (SEM) analysis

SEM (JEOL, Japan) was used to reveal the effect of the algal extract on the *K. pneumoniae* morphology as previously explained [28].

#### Antibiofilm action of *A. maxima* using crystal violet and qRT-PCR

Crystal violet assay was employed to unveil the potential antibiofilm impact of the algal extract as previously explained [29, 30] (supplementary file).

For the molecular investigation of the potential antibiofilm impact of the algal extract on the expression of the biofilm genes (*tre*C, *fim*A, and *mrk*A) in the tested *K. pneumoniae* isolates, qRT-PCR was employed as previously reported [31] (supplementary file). The primer sequences are mentioned in Table S3 using *23srRNA* as a housekeeping gene [28].

### In vivo antibacterial action of *A. maxima*

#### Animals and experimental design

Twenty male albino mice with weights ranging between 21 and 25 g were purchased from Abo Rawash farm in Cairo, Egypt. They were housed for a week under standard conditions of temperature (22–25 °C), humidity (40–60%), and 12-h light–dark cycles. Animals were kept in plastic cages with wire mesh covers and received a standard pellet diet and filtered water throughout the experimental period. The experiments were carried out following the guidelines of the Animal Ethics Committee in the Faculty of Pharmacy at Tanta University, Tanta, Egypt (approval no. TP/RE/4/24 p-002).

A representative colistin-susceptible *K. pneumoniae* isolate was cultured overnight and then adjusted to 4–6 × 10^7^ CFU/mL. Animals were anesthetized and pneumonia was induced by intranasal dripping of 40 μL of *K. pneumoniae* suspension. After 24 h, animals were separated into four groups (n = 5) as follows: group I served as negative control (not infected and received 0.9% saline), and group II was infected and treated with intranasal 0.9% saline to serve as positive control group. Group III was infected and treated with intranasal colistin solution (61.5 mg/kg) to serve as a standard drug-treated group. Group IV was infected and treated with intranasal algal suspension (30 mg/kg). All groups were treated for three consecutive days [32]. On the 4th day, all animals were euthanized, and lungs were extracted and subjected to histopathological and immunohistochemical investigation.

#### Histopathological and immunohistochemical staining

After sacrificing mice, 10% buffered formalin was used for the fixation of extracted lung specimens for 24 h. After dehydration of lung specimens with ethanol, they were embedded in paraffin wax. Preparation of five-micrometer thick sections was made and stained with both hematoxylin and eosin (H&E) [33, 34] and Masson’s trichrome stain for collagen fibers detection. Immunohistochemistry employed monoclonal antibodies for the detection of interleukin six (IL-6) and tumor necrosis factor-alpha (TNF-α) [35, 36].

### Statistical analysis

The results were demonstrated as mean ± standard deviations (SD) as the tests were performed in triplicate. Data’s statistical analysis was executed through one-way analysis of variance (ANOVA) using computerized Graph Pad Prism 8. The level of significance was considered at *p* < 0.05.

## Results

### Antibiotic susceptibility testing of the carbapenem-resistant *K. pneumoniae* isolates and detection of carbapenem-resistance genes

**Fig. 1:**
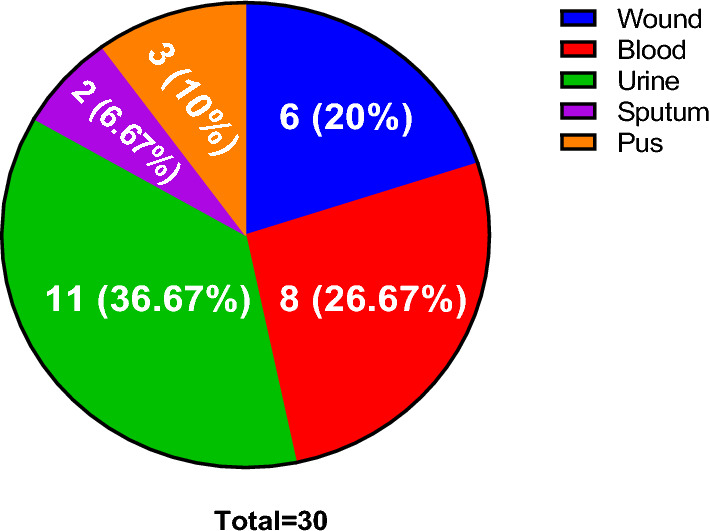
Pie chart revealing the different specimen types

Carbapenem-resistant *K. pneumoniae* isolates were collected from blood, wound, urine, sputum, and pus (Fig. 1). The recovered isolates were collected from 14 (46.67%) males and 16 (53.33%) females.

**Fig. 2:**
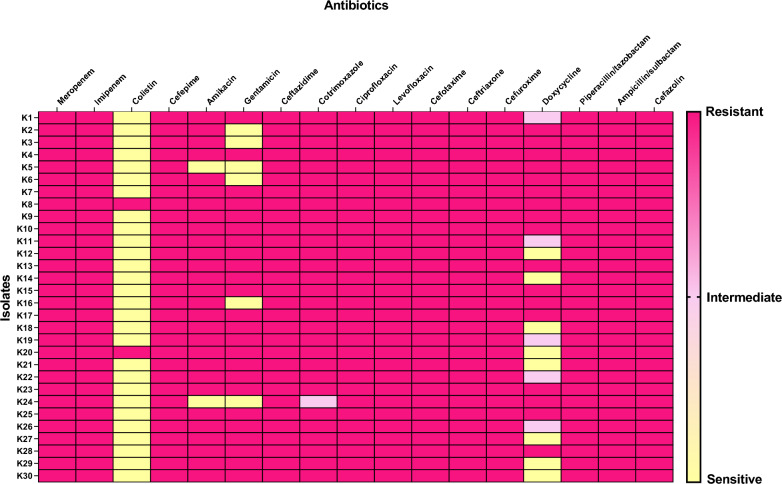
Heat map revealing the antibiotic susceptibility of the tested isolates

All 30 (100%) tested isolates were resistant to meropenem and imipenem by broth microdilution method using meropenem. The carbapenem resistance was confirmed by the disk diffusion method using disks of both meropenem and imipenem. All isolates were resistant to ampicillin/sulbactam, piperacillin/tazobactam, ciprofloxacin, levofloxacin, cefazolin, cefuroxime, cefotaxime, ceftriaxone, ceftazidime and cefepime. Only two isolates (6.67%) were resistant to colistin while 24 (80%) isolates were resistant to gentamicin. Twenty-eight (93.33%) isolates were resistant to amikacin while twenty-nine isolates (96.67%) were resistant to sulfamethoxazole/trimethoprim. Moreover, 17 isolates (56.67%) showed resistance against doxycycline (Fig. 2).

### PCR

**Fig. 3:**
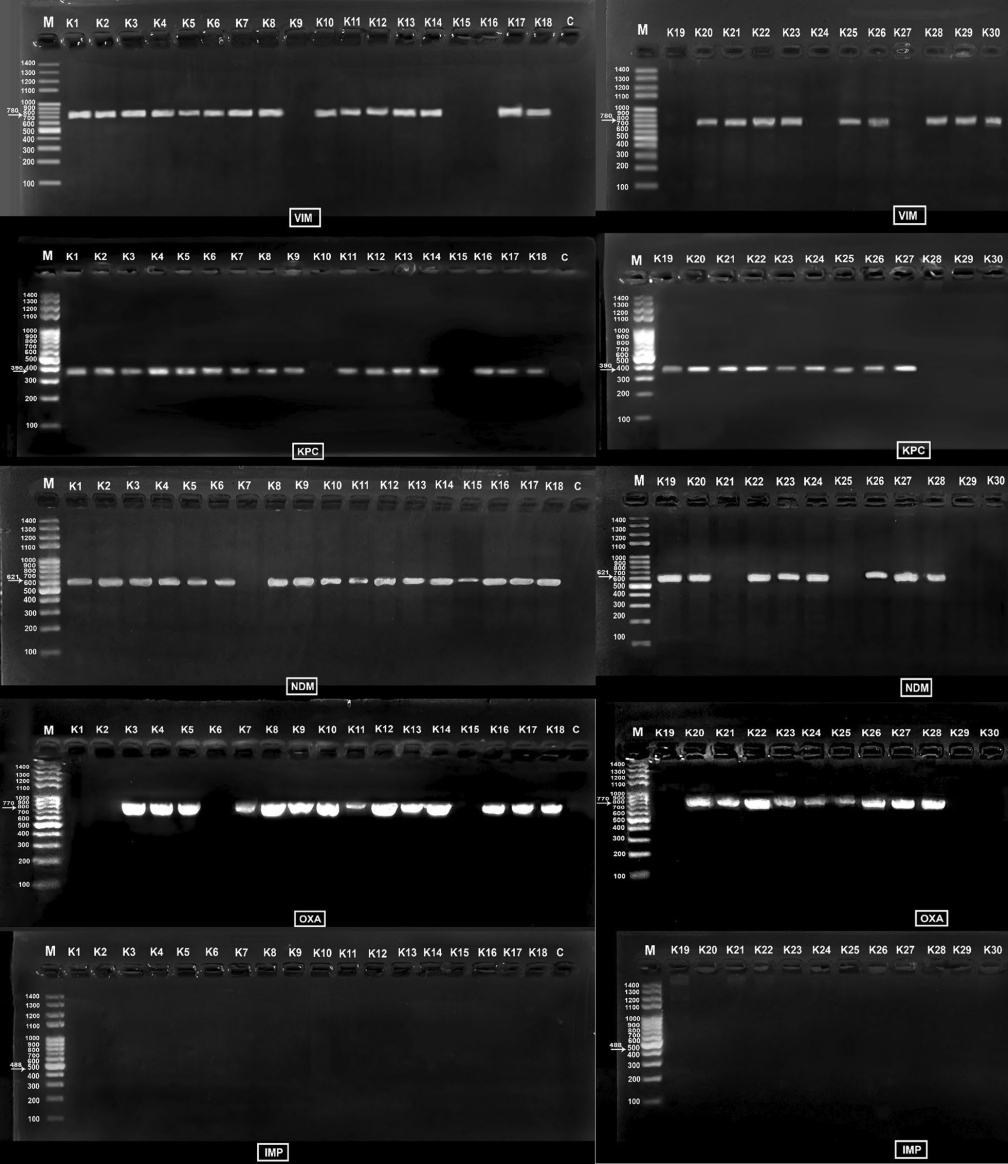
Electropherogram of the PCR amplified products

The PCR results (Table S4 and Fig. 3) revealed that the most predominant carbapenemase resistance genes were KPC and NDM-1. Twenty-five isolates (83.33%) harbored both the KPC and NDM-1 genes. The second prevailing gene was VIM in 24 (80%) of the isolates. OXA-48 was also common being carried by 23 (76.67%) of the isolates. However, IMP was absent in all the tested isolates.

### LC/MS

**Fig. 4:**
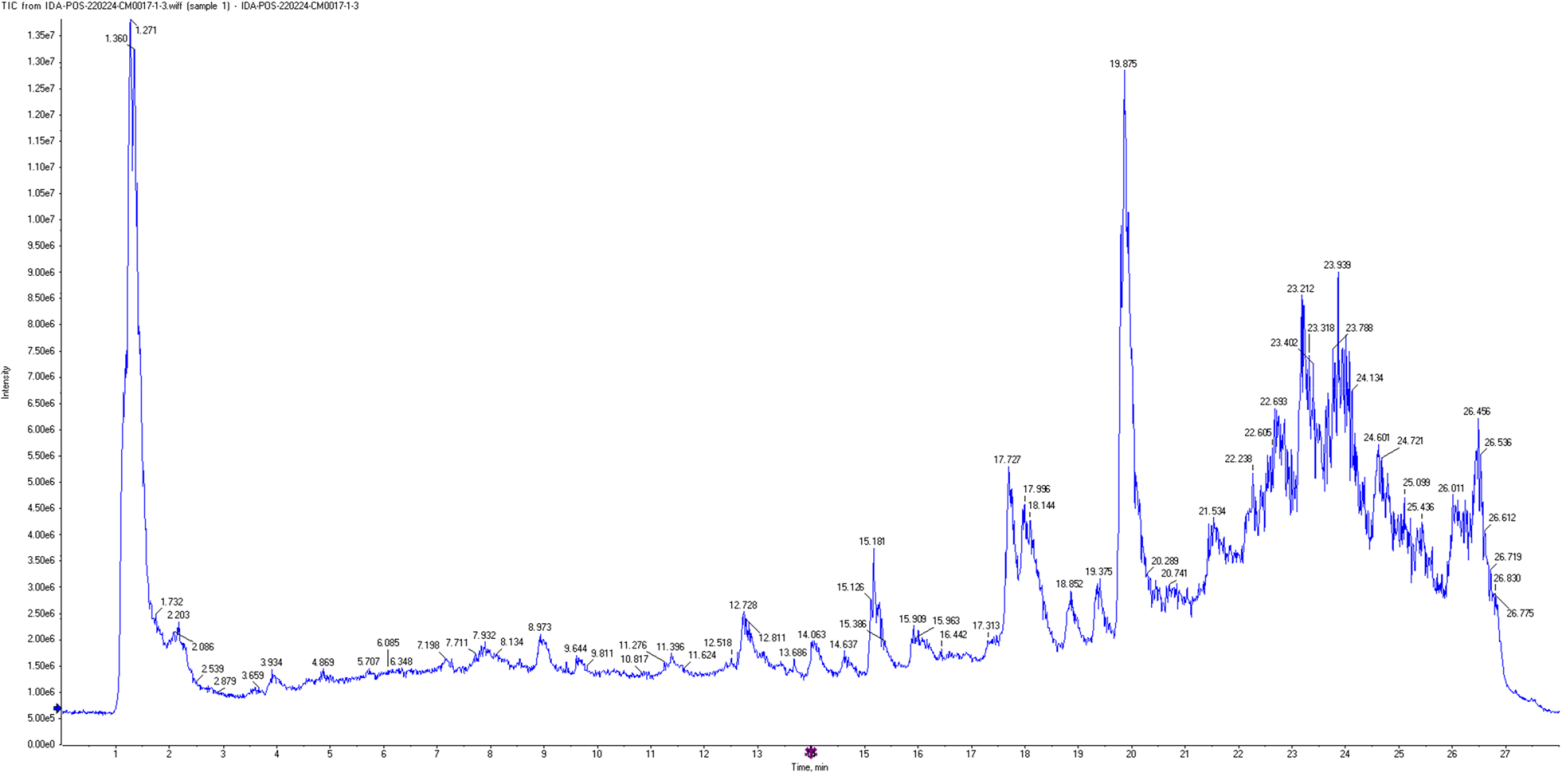
The total ion chromatograms (TIC) of *A. maxima* algal extract positive ion mode

A total of 23 compounds were detected in *A. maxima* extract using LC–ESI–MS/MS (positive mode). The main substances were amino acids, indoles, alkaloids, terpenoids, fatty acids, flavonoids, and carboxylic acids. The metabolite profile is presented in Table 1, while Fig. 4 shows the total ion chromatograms (TIC) of *A. maxima* algal extract positive ion mode.

**Table 1:** Phytochemical profiling of *A. maxima* algal extract by LC–MS/MS analysis (positive mode ESI)

Peak NO	Rt (min)	Precursor *m/z*	Metabolite name	Structure	Adduct ion	MS/MS	Molecular formula
1	1.08	146.16	Spermidine		[M + H]^+^	58.06, 72.08, 84.08, 112.11, 129.13	C_7_H_19_N_3_
2	1.27	162.11	Carnitine		[M + H]^+^	60.08, 85.02, 102.09, 103.03	C_7_H_15_NO_3_
3	1.35	139.05	Urocanic acid		[M + H]^+^	66.03, 93.04, 121.04	C_6_H_6_N_2_O_2_
4	1.58	86.09	Piperidin		[M + H]^+^	56.06, 57.05, 69.06	C_5_H_11_N
5	1.73	124.03	Nicotinic acid		[M + H]^+^	78.03, 80.04, 106.02	C_6_H_5_NO_2_
6	2.08	130.04	Pyroglutamic acid		[M + H]^+^	56.04, 84.04	C_5_H_7_NO_3_
7	2.54	190.07	N-Acetylglutamate		[M + H]^+^	84.04, 130.04, 148.06, 172.06	C_7_H_11_NO_5_
8	2.87	166.08	L-(-)-Phenylalanine		[M + H]^+^	77.03, 103.05, 120.08	C_9_H_11_NO_2_
9	3.93	298.09	5'-methyl thioadenosine		[M + H]^+^	119.03, 136.06, 145.03, 163.04	C_11_H_15_N_5_O_3_S
10	4.87	245.12	Pyrrolo[1,2-a]pyrazine-1,4-dione, hexahydro-3-(phenylmethyl)-		[M + H]^+^	70.06, 72.08, 98.06, 137.07, 169.13	C_14_H_16_N_2_O_2_
11	5.7	116.07	L-proline		[M + H]^+^	70.06, 75.03, 81.03	C_5_H_9_NO_2_
12	6.08	211.14	Cyclo (leucyloprolyl)		[M + H]^+^	70.06, 86.09, 98.06, 114.09, 154.07, 183.14	C_11_H_18_N_2_O_2_
13	7.19	211.09	Sinapyl alcohol		[M + H]^+^	211.09, 193.08, 165.09, 151.04, 119.08, 91.05	C_11_H_14_O_4_
14	7.93	197.11	Loliolide		[M + H]^+^	79.05, 107.08, 133.10, 161.09, 179.10	C_11_H_16_O_3_
15	8.13	146.06	3-formylindole		[M + H]^+^	65.02, 91.04, 118.06	C_9_H_7_NO
16	8.93	163.11	Nicotine		[M + H]^+^	53, 57, 79, 91, 105, 115, 120, 128, 132, 135, 145, 163	C_10_H_14_N_2_
17	12.73	269.02	Formononetin		[M + H]^+^	116, 125,167, 191, 209, 269	C_16_H_12_O_4_
18	15.18	223.20	Anthracene-9-carboxylic acid		[M + H]^+^	104.95, 121.05, 185.01, 210.92	C_15_H_10_O_2_
19	17.73	285.08	Acacetin		[M + H]^+^	128.06, 207.06, 241.04, 242.05,270.05, 285.07	C_16_H_12_O_5_
20	19.87	279.16	Dibutyl phthalate		[M + H]^+^	150.02, 149.02, 121.02	C_16_H_22_O_4_
21	23.21	403.23	Acetyl tributyl citrate		[M + H]^+^	403.23, 361.21, 329.15, 259.15, 185.07, 157.01, 139.00, 129.01, 111.00	C_20_H_34_O_8_
22	23.9	455.38	Oleanonic acid		[M + H]^+^	455.34, 437.33, 359.25, 205.19, 147.12, 121.09, 95.08	C_30_H_46_O_3_
23	26.45	317.11	3 3′ 4' 5-Tetrahydroxy-7-methoxy flavone		[M + H]^+^	81.09, 177.00, 19,514, 282.02, 317.04	C_16_H_12_O_7_

### *A. maxima*’s extract antibacterial and antibiofilm activities (in vitro)

The MICs for the *A. maxima* algal extract against the tested carbapenem-resistant *K. pneumoniae* isolates ranged from 500 to 1000 μg/mL (Table S5).

**Fig. 5:**
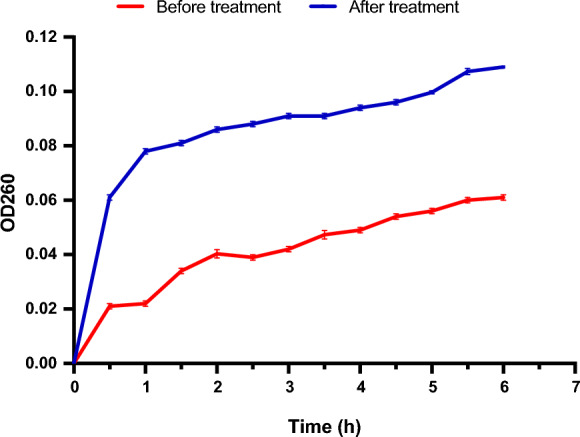
A chart revealing a significant decrease in the membrane integrity of the tested *K. pneumoniae* isolate represented by the significant increase in the absorbance

Exploring the cell membrane integrity of *K. pneumoniae* isolates before and after treatment with 0.5 MIC of the algal extract revealed a substantial decrease (*p* < 0.05) in the cell membrane integrity of all treated isolates. Figure 5 exhibits an interpretative example because the absorbance of DNA and RNA was remarkably higher (*p* < 0.05) after treatment with the algal extract. This means that the cell membrane integrity was notably declined (*p* < 0.05) after treatment.

**Fig. 6:**
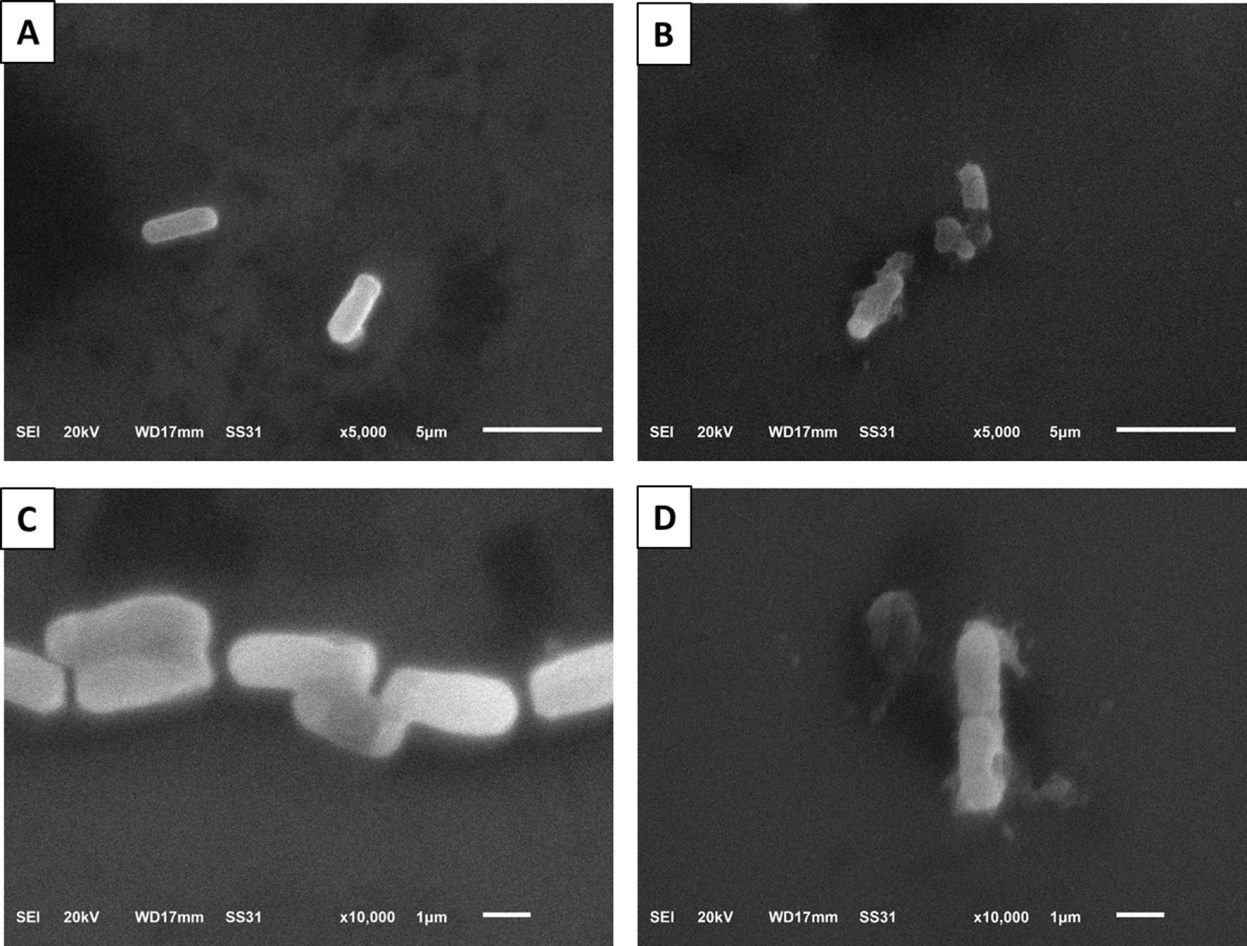
Scanning electron micrograph revealing the cell morphology of *K. pneumoniae* isolate before (**A** and **C**) and after (**B** and **D**) treatment

SEM analysis of the cell morphology of the tested isolates before and after being treated with the algal extract reveals a distortion and damage in the treated bacterial cells induced by *A. maxima* algal extract (Fig. 6).

The antibiofilm activity of the algal extract was elucidated using crystal violet assay. The *A. maxima* algal extract inhibited biofilm formation in 68. 75% of the biofilm forming isolates (Figure S1).

**Fig. 7:**
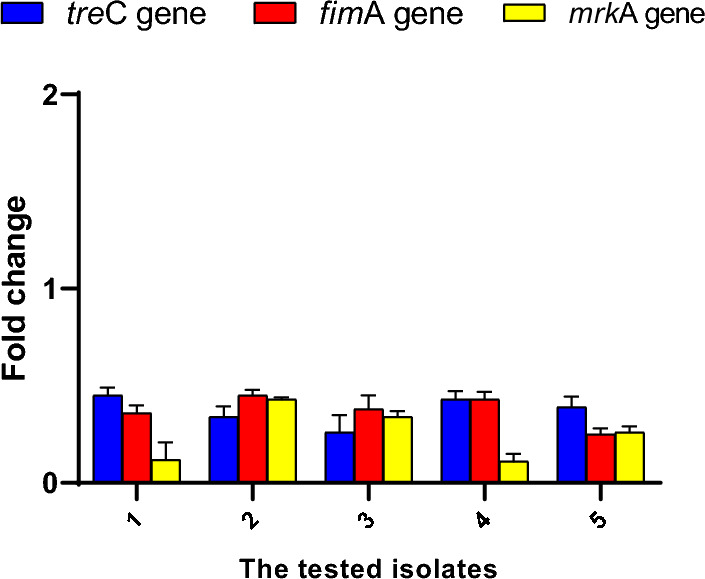
The impact of the algal extract on the gene expression of the biofilm genes

Thus, qRT-PCR was employed to reveal the impact of the *A. maxima* algal extract on the gene expression of the biofilm encoding genes to study its effect at the molecular level. Interestingly, the *A. maxima* algal extract was found to downregulate the biofilm genes in 50% of the isolates (Fig. 7).

### In vivo pneumoniae model

**Fig. 8:**
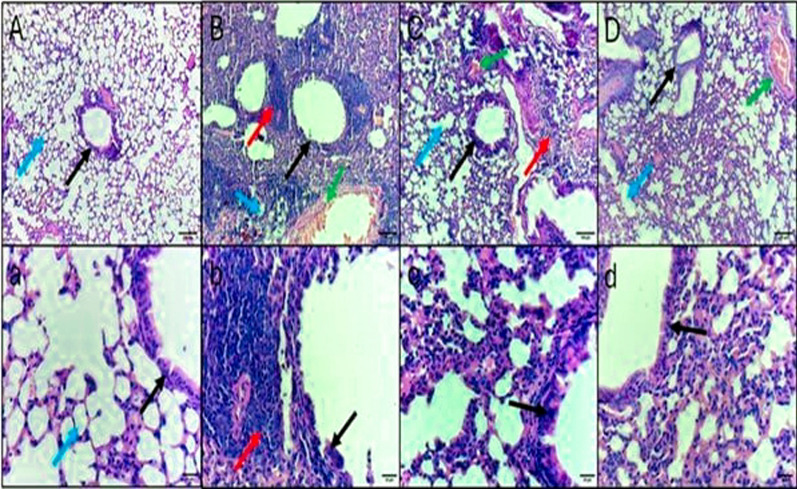
H&E staining of lung sections. **A**, **a** Control negative group, showing normal pulmonary architecture with alveoli that are surrounded and separated from each other by thin interalveolar septa, bronchiole, and bronchial arteriole. **B**, **b** Control positive group, showing distortion in the pulmonary architecture, epithelial damage of the lung bronchi, small sized alveoli due to thickening of interalveolar septa, mononuclear cellular infiltration in septa and around the bronchi, and thick congested pulmonary arteriole. **C**, **c** Standard treatment group, showing partial restoration of pulmonary architecture with larger alveoli separated by thinner interalveolar septa, normal bronchi and bronchial arteriole, and minimal inflammatory cells infiltration. **D**, **d** Test treatment group, showing partial restoration of pulmonary architecture with larger alveoli separated by thinner interalveolar septa, normal bronchi and bronchial arteriole, and no inflammatory cells infiltration. Black arrow: bronchiole, blue arrow: alveoli, red arrow: Inflammatory cells infiltration, green arrow: pulmonary arteriole. Upper raw original magnification 10 × , lower raw 40 × and scale bar 100 µm and 20 µm, respectively

**Fig. 9:**
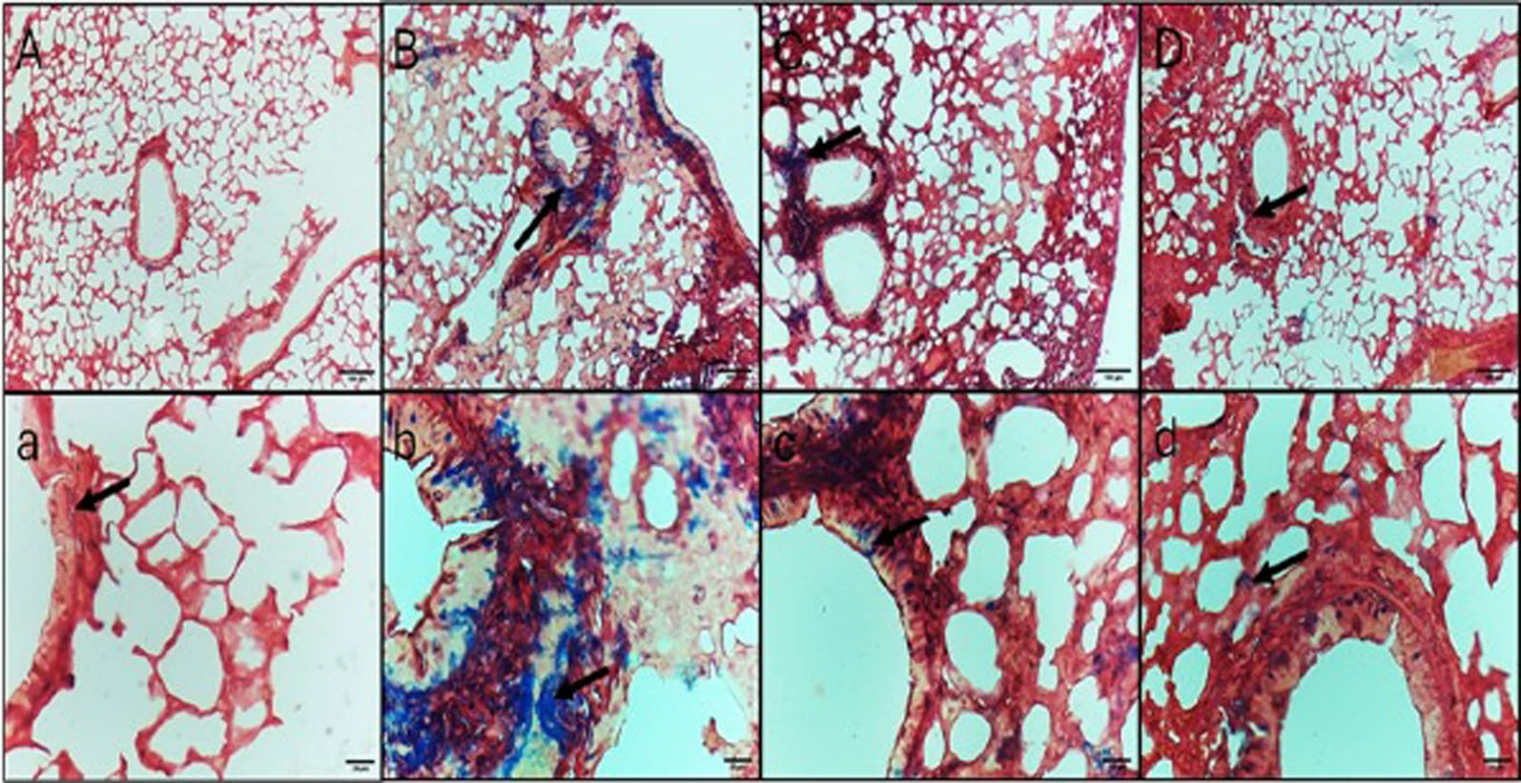
Masson’s trichrome staining of lung sections **A**, **a** Control negative group, showing no fibrosis except few spots in the bronchial wall. **B**, **b** Control positive group, showing fibrosis in the bronchi walls and some alveolar septa. **C**, **c** Standard treatment group, showing less fibrosis in the bronchi walls. **D**, **d** Test treatment group, showing minimal fibrosis. Black arrow: fibrosis. Upper raw original magnification 10 × , lower raw 40 × and scale bar 100 µm and 20 µm, respectively

H&E and Masson’s trichrome staining micrographs of the lung tissue sections are revealed in Figs. 8 and 9.

**Fig. 10:**
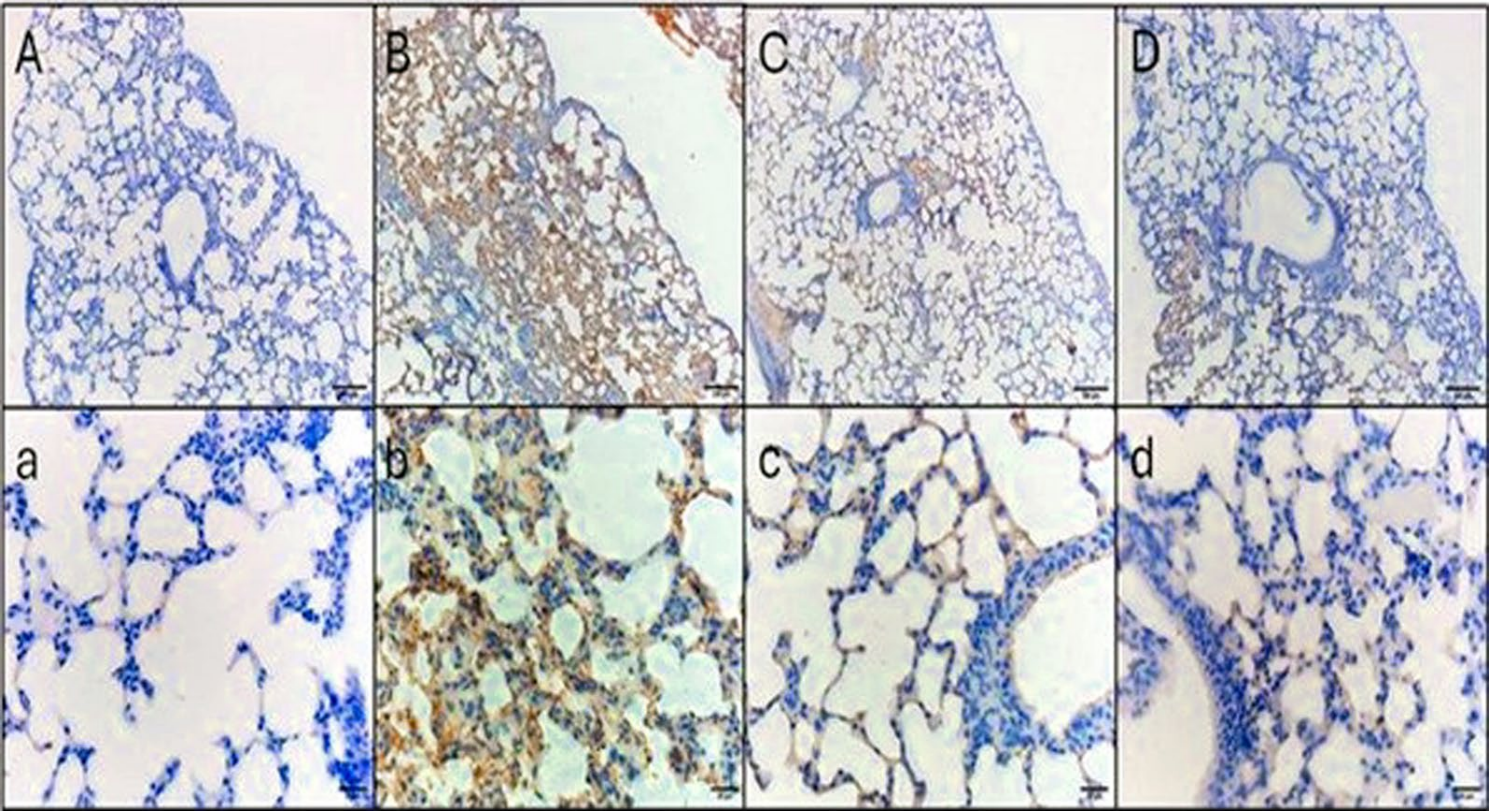
IL-6 immunohistochemical staining of lung tissue sections **A**, **a** Control negative group, indicating no immunoreactivity in the alveolar walls. **B**, **b** Control positive group, revealing fierce cytoplasmic immunoreactivity in the alveolar walls. **C**, **c** Standard treatment group, showing mild cytoplasmic immunoreactivity. **D**, **d** Test treatment group, showing mild cytoplasmic immunoreactivity. Upper raw original magnification 10 × , lower raw 40 × and scale bar 100 µm and 20 µm, respectively

**Fig. 11:**
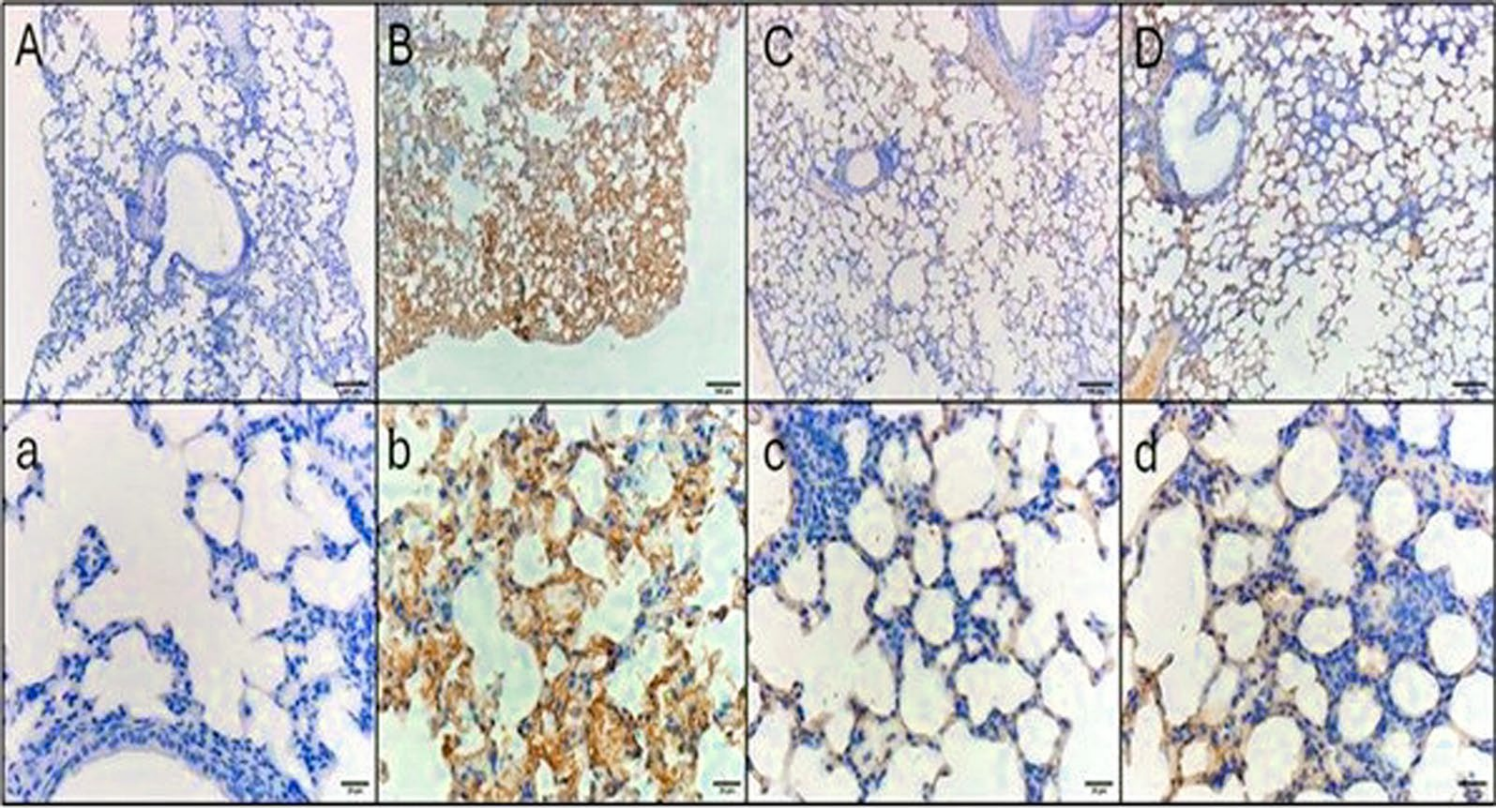
TNF-α immunohistochemical staining micrographs of lung tissue sections of **A**, **a** Control negative group, indicating no immunoreactivity in the alveolar walls. **B**, **b** Control positive group revealing fierce cytoplasmic immunoreactivity in the alveolar walls. **C**, **c** Standard treatment group, showing mild cytoplasmic immunoreactivity. **D**, **d** Test treatment group, showing mild cytoplasmic immunoreactivity. Upper raw original magnification 10 × , lower raw 40 × and scale bar 100 µm and 20 µm, respectively

Figures 10 and 11 reveal the IL-6 and TNF-α immunohistochemical staining micrographs of the lung tissue sections of the different experimental groups.

**Fig. 12:**
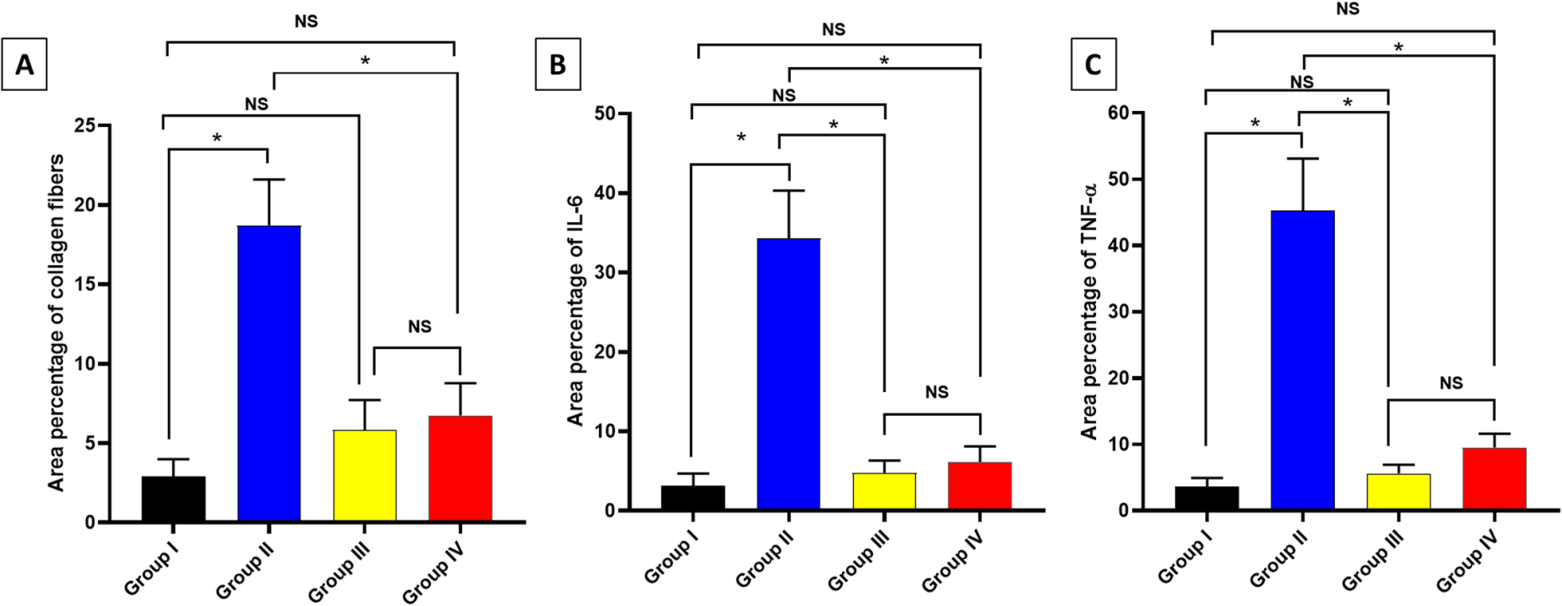
Area percentages of the **A** collagen fibers, **B** IL-6, and **C** TNF-α. The single asterisk demonstrates a substantial change (*p* < 0.05). The abbreviation (NS) means a non-significant change (*p* > 0.05)

The area percentage of the collagen fibers revealed by Masson’s trichrome stain and the area percentage of IL-6 and TNF-α revealed by the immunohistochemical studies are demonstrated in Fig. 12

## Discussion

*K. pneumoniae* is considered one of the most predominant Gram-negative bacteria which results in serious nosocomial infections. The situation often exacerbate when these infections are caused by MDR pathogen, which restricts the available therapy options [37]. Resistance to carbapenems among the family *Enterobacteriaceae*, especially *K. pneumoniae*, has been emerged as a global concern in the last decade. This is due to the rapid outbreak of carbapenemase producing strains which represent a fatal threat to the public health [38]. Carbapenemases are considered the primary factor which lead to the incidence of extensive resistance and their acquisition and dissemination which possibly warns a pan-drug resistance in the near future [39]. Previous studies documented firm relationships between hospitals and acquisition of carbapenem-resistant bacteria [40–42].

In this study, all tested isolates exhibited resistance to two carbapenems (meropenem and imipenem). Former studies have depicted similar results in Egypt. Mohamed Gandor et al. reported that 100% of the tested *K. pneumoniae* isolates were resistant to imipenem and meropenem [43]. Another Egyptian study in Mansoura University Hospitals showed 42 (33.6%) of 125 isolates were resistant to carbapenems [44]. Moreover, another previous investigation in Suez Canal University Hospitals showed a prevalence of 44.3% of carbapenem-resistant *K. pneumoniae* isolates [45]. In addition, previous study from the Egyptian National Cancer Institute reported a lower incidence of 13.9% carbapenem resistance among *K. pneumoniae* [46]. A study conducted in USA reported that 83% of isolates were carbapenem-resistant [47]. This high incidence of carbapenem-resistant *K. pneumoniae* observed in the current study may be credited to the heavy usage of carbapenems as an empirical therapy in intensive care units and hospital wards at these institutions.

The *bla*_KPC_ and *bla*_OXA-48_ genes were the most prevalent carbapenemase genes among carbapenem-resistant *K. pneumoniae* [48]. However, new Delhi metallo-β-lactamase (NDM) has been previously proclaimed to be prevailing during the latter decade, which accounted for 11% and 30% of cases of carbapenem resistance in Europe [49] and China [50], respectively. Moreover, *bla*_NDM_ has been the leading carbapenemase in pediatric patients in China and several other countries [51]. Results of this study are disturbing as molecular studies showed a double or triple carbapenemase gene combination in most isolates (*bla*_NDM-1_, *bla*_KPC_, *bla*_OXA-48_) where 90% of the *K. pneumoniae* isolates encoded at least two carbapenemase genes. Both *bla*_KPC_ and *bla*_NDM-1_ were the most predominant carbapenem resistance genes in the current study as they were identified in 83.3% of the tested isolates. The second prevailing gene was *bla*_VIM_ which was harbored by 80% of the 30 isolates. The third in line was *bla*_OXA-48_ which was identified in 76.6% of the isolates. Remarkably, neither of the tested 30 isolates harbored the *bla*_IMP_ gene. A former study reported that *bla*_KPC_ gene was the most predominant gene among the tested 42 carbapenem-resistant *K. pneumoniae* isolates. This agrees with a published study in Bahrain reporting that 95.8% of the isolates were harboring *bla*_NDM-1_. A Turkish study reported that carbapenem-resistant *K. pneumoniae* isolates had *bla*_NDM-1_ and *bla*_OXA-48_ in 38.9% and 81.05% of the isolates respectively. A Saudi Arabian study reported that 80.9% of carbapenem-resistant *K. pneumoniae* isolates encoded triple resistance genes (*bla*_KPC_, *bla*_NDM-1_, and *bla*_OXA-48_) meanwhile 19.04% of them encoded double resistance genes (*bla*_KPC_ and *bla*_OXA-48_) or (*bla*_NDM-1_, *bla*_OXA-48_). Additionally, previous studies reported that carbapenem-resistant *K. pneumoniae* isolates recovered from different hospitals in Saudi Arabia and other countries in the Arabian Peninsula were reported to be *bla*_OXA-48_ and bla_NDM_ carriers [52, 53].

Such problematic bacteria that represent a great barrier to the clinical treatment of multidrug-resistant infections need to be continuously investigated to explore novel therapeutic options. Thus, here we aimed at elucidating the potential antibacterial action of *A. maxima* algal extract against the studied bacterial isolates.

*A. maxima* (spirulina) has been used for a long time as an additive in healthy food. Subsequently, the commercial production of spirulina has acquired a global interest because of its various benefits. Spirulina could inhibit the growth of multiple microorganisms owing to its high content of bioactive constituents that exhibit antimicrobial activity [54] like phycocyanin and polysaccharides that harm bacterial cell walls and cause oxidative stress that break down bacterial DNA and proteins. Additionally, it produces an alkaline antioxidant-rich environment that inhibits infections and promotes the growth of good bacteria. Thus, spirulina is a good choice as an antibacterial agent in foods and supplements [14]. In the current study, the LC/MS analysis revealed different phytochemicals such as flavonoids, terpenoids, fatty acids, indoles, and carboxylic acids.

Our study findings revealed that *A. maxima* extract had antibacterial activity against carbapenem-resistant *K. pneumoniae* isolates with MIC values ranging from 500 to 1000 µg/mL. This is lower than that reported by a previous study which reported that the MIC values of spirulina ethanolic extract to be 1000–2000 µg/mL [55]. Nainangu et al. [56] reported that the methanol-chloroform extracts of *Oscillatoria* sp. SSCM01 exhibited a higher antibacterial activity than *Phormidium* sp. SSCM02. *Oscillatoria* sp. SSCM01 exhibited higher inhibition activity of bacterial growth at 31.2 μg/mL and 7.8 μg/mL as MIC value against *Staphylococcus aureus*, *Salmonella typhi*, respectively, and for *Candida albicans* at 125 μg/mL.

Here, we investigated the potential effect of the *A. maxima* algal extract on the membrane integrity and bacterial morphology using SEM. Interestingly, we found that there was a notable decline in the bacterial cell membrane integrity and distortion of the bacterial cells after treatment with the *A. maxima* algal extract which had a detrimental effect on the bacterial cells due to leakage of the cytoplasmic content. Similar results were revealed by Alshuniaber et al. [21].

The biofilm-forming capability of the isolates, a major aspect of *K. pneumoniae*’s pathogenicity, was also explored. Biofilm is a major virulence factor that contributes hugely to the spread of antibiotic resistance among bacterial isolates, particularly, *K. pneumoniae* [57]. Biofilms enable the bacterial cells to attach to the host surfaces, escape the immune system, and resist antibiotics, which induce a significant risk for the host [58]. Here, the antibiofilm action of the *A. maxima* algal extract was investigated using crystal violet assay. We found that the *A. maxima* algal extract inhibited biofilm formation in 68.75% of the biofilm-forming isolates. Also, the algal extract was found to downregulate the biofilm genes (*tre*C, *fim*A, and *mrk*A) in 50% of the isolates. *K. pneumoniae* isolates form biofilms with the aid of type 1 and type 3 pili. The *fim*A and *mrk*A genes encode the major fimbrial subunits [59]. Moreover, the *tre*C gene affects biofilm formation by modulating the production of capsular polysaccharides [60]. Consistent with the findings from previous related studies [61, 62], the current investigation revealed a notable inhibitory effect on the biofilm formation ability of the tested isolates after treatment with *A. maxima* algal extract.

A substantial antibacterial action of *A. maxima* was observed in the employed pneumonia model in mice. H&E staining, Masson’s trichrome staining, and immunohistochemistry of lung tissue sections showed that the *A. maxima* algal extract had a curing effect that was comparable to colistin, with mild cytoplasmic immunoreactivity and minimal fibrosis. A previous study reported that spirulina had mitigated the lung injury induced by radiation in rats [63]. *A. maxima* is suggested to stimulate the immune system and has anti-inflammatory and antioxidant activity.

In autoimmune illnesses and other immunological and inflammatory processes, IL-6 stimulates T-cell differentiation and B-cell proliferation, which can intensify chronic inflammatory responses. Increased IL-6 levels are correlated with the severity of disorders such as rheumatoid arthritis and autoimmune encephalitis, making IL-6 a crucial focus in these conditions [64]. As a main originator of inflammatory signaling, TNF-α triggers a cascade that includes IL-6 and other cytokines, particularly in acute and chronic inflammatory disorders such as inflammatory bowel disease and psoriasis [65]. This is attributed to its bioactive compounds which belong to different chemical classes that are reported to have many pharmacological activities and these classes include indoles [66], monoterpenoids [67], phenolics [67], and carboxylic acids [68]. A previous study performed by Manivannan et al. [69] revealed the anti-inflammatory action of compounds isolated from cyanobacteria which is comparable to our study.

## Conclusion

In addition to its known nutritional properties, our in vivo and in vitro study results revealed potential antibacterial and antibiofilm activities of *A. maxima* against the tested carbapenem-resistant *K. pneumoniae* isolates. Moreover, *A. maxima* markedly decreased the inflammation that was triggered by the induced infection. Further clinical studies are recommended to confirm our results to study their safety and efficacy in humans as effective alternative natural products. Accordingly, these friendly algae could have the potential to be considered in future investigations as an important nutraceutical supplement to combat infections caused by problematic multidrug-resistant bacteria.

## Supplementary Information

Supplementary material 1.

## Data Availability

No datasets were generated or analysed during the current study.
