# Supplementary material for: Unveiling the potential of spirulina algal extract as promising antibacterial and antibiofilm agent against carbapenem-resistant Klebsiella pneumoniae: in vitro and in vivo study
Source: Microb Cell Fact. 2025 Jan 5;24:7. doi: 10.1186/s12934-024-02619-3 (PMC11700449; doi:10.1186/s12934-024-02619-3)
Supplement: Supplementary file 1 — Supplementary material 1. [file 12934_2024_2619_MOESM1_ESM.docx]

**Collection of bacterial isolates**

**Table S1.** Samples’ type of bacterial isolates in this study

| **Isolate number** | **Sample type** |  |
| --- | --- | --- |
|  |  |  |
|  |  |  |
| K1 | Wound |  |
| K2 | Blood |  |
| K3 | Blood |  |
| K4 | Pus |  |
| K5 | Pus |  |
| K6 | Wound |  |
| K7 | Urine |  |
| K8 | Wound |  |
| K9 | Blood |  |
| K10 | Wound |  |
| K11 | Urine |  |
| K12 | Urine |  |
| K13 | Blood |  |
| K14 | Pus |  |
| K15 | Sputum |  |
| K16 | Urine |  |
| K17 | Urine |  |
| K18 | Wound |  |
| K19 | Urine |  |
| K20 | Urine |  |
| K21 | Blood |  |
| K22 | Sputum |  |
| K23 | Blood |  |
| K24 | Urine |  |
| K25 | Urine |  |
| K26 | Urine |  |
| K27 | Blood |  |
| K28 | Urine |  |
| K29 | Wound |  |
| K30 | Blood |  |

**Polymerase chain reaction (PCR)**

*K. pneumoniae* in this study were subjected to PCR assay to detect any of the five carbapenem resistance genes: *bla*_VIM_, *bla*_IMP_, *bla*_NDM-1_, *bla*_KPC_ and *bla*_OXA-48_ as previously reported. The primer set sequence for each gene is shown in Table S2.

QIAprep® spin Miniprep kit (Qiagen, Germany) was utilized for plasmid extraction. In this procedure, a volume of one milliliter of overnight-cultured bacterial growth underwent centrifugation at a speed exceeding 8000 rpm for three minutes at room temperature then resuspension of the formed pelleted bacterial cells in 250 μL Buffer P1. 250 μl of Buffer P2 was added to the tube then the tube was inverted 4-6 times till the solution became clear. Subsequently, 350 μL of Buffer N3 was added and mixed immediately by gently inverting the tube 4-6 times and then centrifuged at speed of 13000 rpm for 10 minutes. 800 μL of the formed supernatant was transferred to the spin column and then centrifuged at speed of 13000 rpm for one minute. The spin column was washed by addition of 0.75 ml Buffer PE and then centrifuged for one minute. Finally, DNA was eluted by addition of 50 μl of Buffer EB to the spin column in a clean microcentrifugation tube, let stand for one minute then centrifuged for one minute.

Dilution of each primer was done by addition of 10 μl of the primer to 90 μl of nuclease free water. In a PCR tube, 12.5 μl of Cosmo Taq Red Master Mix 2x (Willowfort, UK), 1 μl of forward primer, 1 μl of reverse primer, 1 μl of the template DNA and 9.5 μl of nuclease free water were added to obtain a total volume of 25 μl. A PCR tube free of template DNA was utilized as negative control.

PCR was carried out as following: one cycle of initial denaturation at 95 °C for five minutes, then 35 cycles of (denaturation at 95 °C for 30 seconds, annealing for 30 seconds at 52 °C for *bla*_VIM_, *bla*_IMP_ and *bla*_NDM-1_, while at 48°C for 30 seconds for *bla*_KPC_ and 58°C for 30 seconds for *bla*_OXA-48_ and extension at 72°C for one minute) and one cycle of final extension at 72°C for five minutes.

**Table S2.** Primer sequence for the PCR studies

| Amplicon Size (bp) | Sequence | Type | Gene's name |
| --- | --- | --- | --- |
| 780 | 5`… ATTGGTCTATTTGACCGCGTC | F | *bla*_VIM_ |
|  | 5`… TGCTACTCAACGACTGAGCG | R |  |
| 770 | 5`…ATGCGTGTATTAGCCTTATCGGC | F | *bla*_OXa-48_ |
|  | 5`… ACTTCTTTTGTGATGGCTTGGCGCA | R |  |
| 621 | 5`… GGTTTGGCGATCTGGTTTTC | F | *bla*_NDM-1_ |
|  | 5`… CGGAATGGCTCATCACGATC | R |  |
| 488 | 5`… CATGGTTTGGTGGTTCTTGT | F | *bla*_IMP_ |
|  | 5`… ATAATTTGGCGGACTTTGGC | R |  |
| 390 | 5`… CGTTGACGCCCAATCC | F | *bla*_KPC_ |
|  | 5`… ACCGCTGGCAGCTGG | R |  |

**Antibiofilm action**

Isolates were tested for their capability of biofilm formation using microtiter plate assay. In a sterile 96-microtiter plate, A volume of 100 μL of freshly prepared nutrient broth was added to each well followed by addition of 20 μL of overnight bacterial suspension (0.5 McFarland). Positive control contained bacterial culture and nutrient broth while negative control (blank) contained nutrient broth only. Plates were incubated at 37 °C for 24 h then rinsed with 0.9% saline. Remaining bacteria were then resuspended with 100 μl of nutrient broth. In another sterile plate, 100 μL of nutrient broth were transferred to each well. A 100 μL volume of algal aliquot with a concentration of 2000 μg/mL was added to first well then subjected to two-fold serial dilution to challenge bacteria until reaching well 10 followed by addition of 20 μL of the remaining bacterial suspension. Positive control was not treated with algae and contained the broth and bacteria and negative control contained clean broth only. After incubation for 24 h at 37 °C, plates were emptied, rinsed with saline to remove excess free cells then stained with 100 μL of 0.1% crystal violet and let sit for 15 minutes. Plates were washed again by saline to remove excess crystal violet residues then 100 μl of 30% glacial acetic acid was added to solubilize the dye. After incubation for 10 minutes, absorbance was measured using microplate reader (readwell TOUCH, ROBONIK, India) at wavelength 630 nm.

**qRT-PCR studies**

The expression levels of the biofilm genes were detected after treatment with the algal extract using qRT-PCR. Briefly, after centrifugation of the overnight cultures of *K. pneumoniae* isolates (with and without treatment), the pellets were immediately utilized for total RNA extraction using the Purelink® RNA Mini Kit (Thermo SCIENTIFIC, Waltham, USA) as described by the manufacturer. The purified RNA was rapidly retrotranscribed into cDNA using a power cDNA synthesis kit (iNtRON Biotechnology, Korea) as recommended by the manufacturer. qRT-PCR was accomplished using the Rotor-Gene Q 5plex machine (Qiagen, Hilden, Germany). The qRT-PCR was conducted to calculate the fold changes in the expression of the tested genes using the housekeeping gene *23S rRNA* as an endogenous control. The used primers are listed in Table S3. The relative levels of target gene expression were quantified using the 2^−ΔΔCt^ method.

**Table S3.** Primer sequences of the biofilm genes.

| Gene | Primer sequence (5` to 3`) |
| --- | --- |
| *tre*C | F: CCGACAGCGGGCAGTATT  R: CGCCGGATTCTCCCAGTT |
| *fim*A | F: CGGACGGTACGCTGTATTTT  R: GCTTCGGCGTTGTCTTTATC |
| *mrk*A | F: CGGTAAAGTTACCGACGTATCTTGTACTG  R: GCTGTTAACCACACCGGTGGTAAC |
| *23srRNA* | F: ATCGTACCCCAAACCGACAC  R: TTCTCCCGAAGTTACGGCAC |

**Table S4.** PCR results of the carbapenemase genes in the tested isolates

| Isolate | VIM | KPC | NDM-1 | IMP | OXA-48 |
| --- | --- | --- | --- | --- | --- |
| K1 | (+) | (+) | (+) | (-) | (-) |
| K2 | (+) | (+) | (+) | (-) | (-) |
| K3 | (+) | (+) | (+) | (-) | (+) |
| K4 | (+) | (+) | (+) | (-) | (+) |
| K5 | (+) | (+) | (+) | (-) | (+) |
| K6 | (+) | (+) | (+) | (-) | (-) |
| K7 | (+) | (+) | (-) | (-) | (+) |
| K8 | (+) | (+) | (+) | (-) | (+) |
| K9 | (-) | (+) | (+) | (-) | (+) |
| K10 | (+) | (-) | (+) | (-) | (+) |
| K11 | (+) | (+) | (+) | (-) | (+) |
| K12 | (+) | (+) | (+) | (-) | (+) |
| K13 | (+) | (+) | (+) | (-) | (+) |
| K14 | (+) | (+) | (+) | (-) | (+) |
| K15 | (-) | (-) | (+) | (-) | (-) |
| K16 | (-) | (+) | (+) | (-) | (+) |
| K17 | (+) | (+) | (+) | (-) | (+) |
| K18 | (+) | (+) | (+) | (-) | (+) |
| K19 | (-) | (+) | (+) | (-) | (-) |
| K20 | (+) | (+) | (+) | (-) | (+) |
| K21 | (+) | (+) | (-) | (-) | (+) |
| K22 | (+) | (+) | (+) | (-) | (+) |
| K23 | (+) | (+) | (+) | (-) | (+) |
| K24 | (-) | (+) | (+) | (-) | (+) |
| K25 | (+) | (+) | (-) | (-) | (+) |
| K26 | (+) | (+) | (+) | (-) | (+) |
| K27 | (-) | (+) | (+) | (-) | (+) |
| K28 | (+) | (-) | (+) | (-) | (+) |
| K29 | (+) | (-) | (-) | (-) | (-) |
| K30 | (+) | (-) | (-) | (-) | (-) |

**Table S5.** The MIC results of the algal extract against carbapenem-resistant *K. pneumoniae*

| Isolate | MIC for algae (μg/mL) | Isolate | MIC for algae (μg/mL) |
| --- | --- | --- | --- |
| K1 | 500 | K16 | 500 |
| K2 | 500 | K17 | 500 |
| K3 | 500 | K18 | 500 |
| K4 | 500 | K19 | 500 |
| K5 | 500 | K20 | 500 |
| K6 | 500 | K21 | 1000 |
| K7 | 500 | K22 | 1000 |
| K8 | 500 | K23 | 1000 |
| K9 | 500 | K24 | 500 |
| K10 | 500 | K25 | 1000 |
| K11 | 500 | K26 | 500 |
| K12 | 500 | K27 | 500 |
| K13 | 500 | K28 | 1000 |
| K14 | 500 | K29 | 500 |
| K15 | 500 | K30 | 500 |


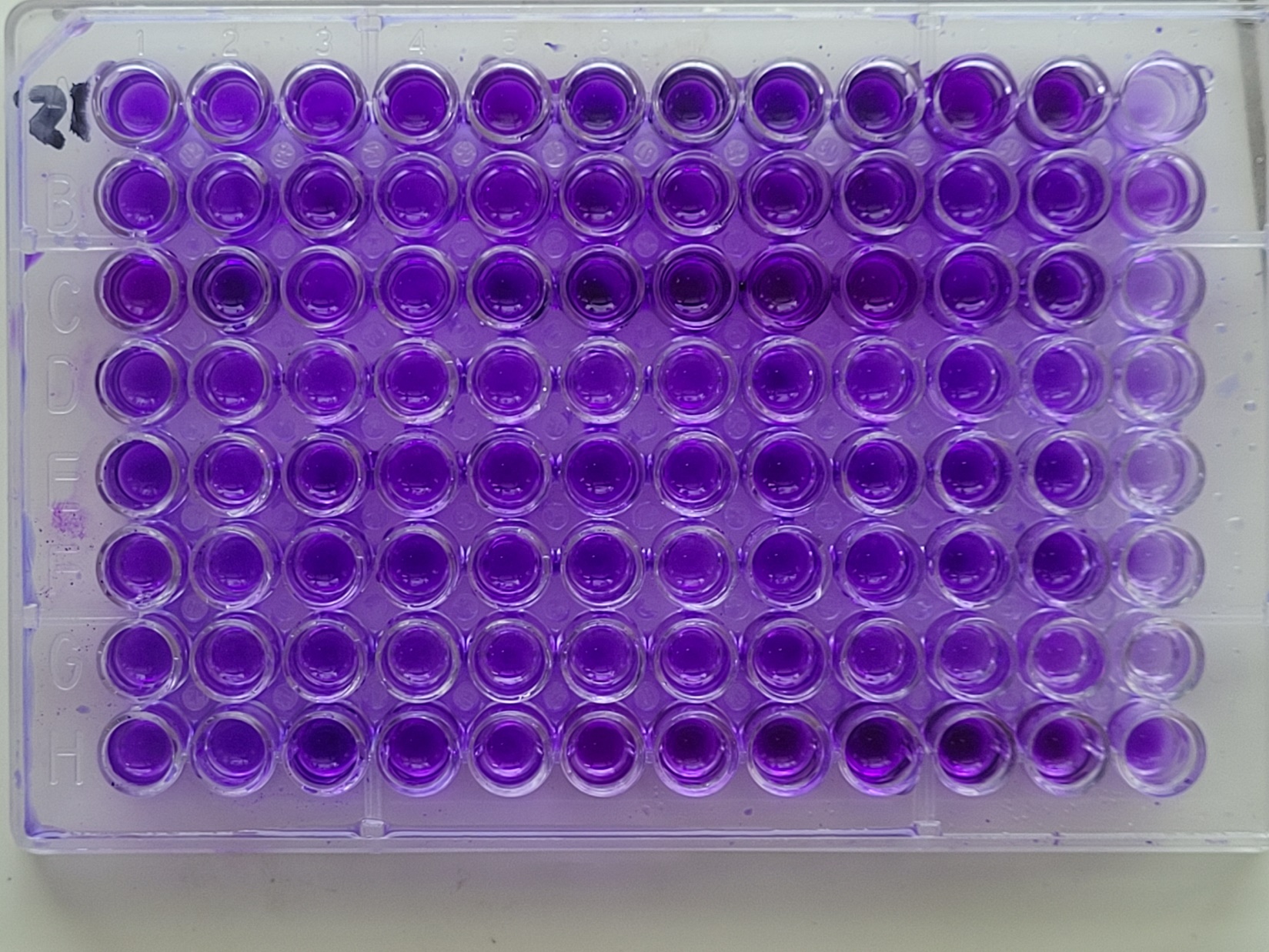

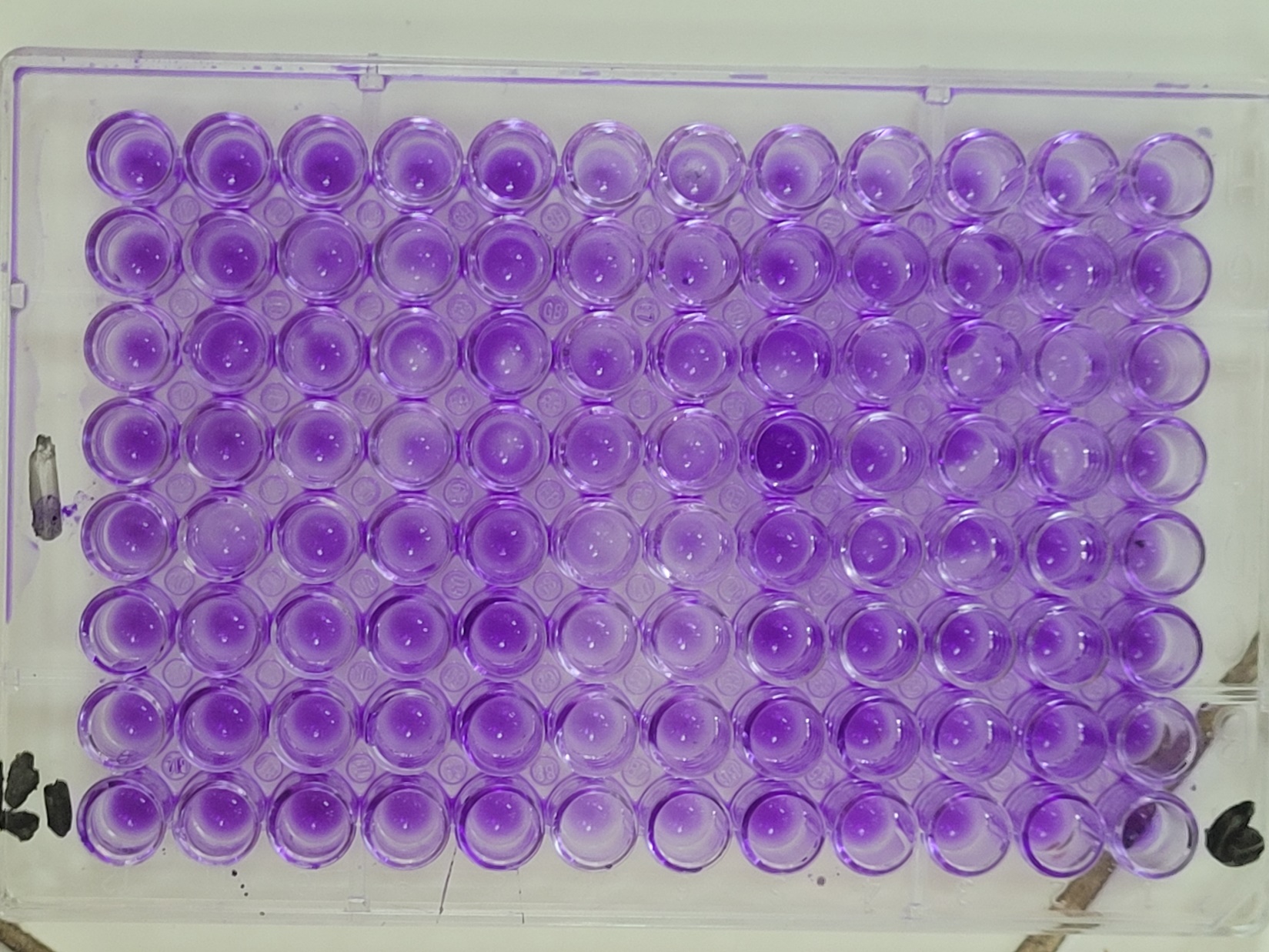


**A**

**B**

**Figure S1.** Impact of the algal extract on biofilm formation A) before treatment and B) after treatment.
